# Inflammatory and Functional Correlates of Depression in Chronic Heart Failure: A Cross-Sectional Study

**DOI:** 10.1192/j.eurpsy.2026.10220

**Published:** 2026-07-31

**Authors:** A. K. Sikora, S. Fedorov

**Affiliations:** 1 Ivano-Frankivsk National Medical University, Ivano-Frankivsk, Ukraine; 2 Mazovian Specialist Hospital “Drewnica”, Ząbki, Poland

## Abstract

**Introduction:**

Depression is prevalent among patients with chronic heart failure (CHF) and is associated with increased morbidity, hospitalization, and reduced quality of life. Inflammation and neurohormonal dysregulation are hypothesized to underlie this association. However, few studies have evaluated depressive symptoms in CHF patients in relation to proinflammatory cytokines, natriuretic peptides, and functional status.

**Objectives:**

To assess the relationship between depressive symptoms and levels of TNF-α, NT-proBNP, and clinical variables including anxiety and quality of life among patients with CHF.

**Methods:**

A total of 120 patients diagnosed with CHF (NYHA II–III) were recruited. Depression was assessed using the Beck Depression Inventory (BDI), and anxiety with the Generalized Anxiety Disorder scale (GAD-7). Quality of life was evaluated using EQ-5D. Serum levels of TNF-α and NT-proBNP were measured by ELISA method. Echocardiographic parameters, demographic variables (age, sex), and lipid profiles were recorded. Correlation and subgroup analyses were conducted to assess associations between biomarker levels and psychological/clinical outcomes.

**Results:**

Depressive symptoms (BDI ≥14) were present in 66.7% of patients. Higher depression scores correlated significantly with elevated TNF-α (r = 0.48, p < 0.01), NT-proBNP (r = 0.42, p < 0.01), and anxiety levels (GAD-7, r = 0.61, p < 0.001). Patients with depression also reported significantly lower EQ-5D index scores (mean 0.56 vs. 0.74, p < 0.001). Women reported higher depressive and anxiety scores than men (p < 0.05), while older patients (>65) showed stronger correlations between NT-proBNP and emotional distress. No significant correlation was observed between lipid levels and depressive symptoms.

**Conclusions:**

Depression in CHF patients is significantly associated with elevated inflammatory and neurohormonal markers, as well as anxiety and reduced quality of life. These findings underscore the importance of psychosomatic assessment in CHF management. Future research should explore integrated treatment strategies targeting both physiological and psychological domains.

**Disclosure of Interest:**

None Declared

